# Discovery, development and application of drugs targeting BCL-2 pro-survival proteins in cancer

**DOI:** 10.1042/BST20210749

**Published:** 2021-09-13

**Authors:** Erinna F. Lee, W. Douglas Fairlie

**Affiliations:** 1 La Trobe Institute for Molecular Science, La Trobe University, Melbourne, Victoria 3086, Australia; 2 Cell Death and Survival Laboratory, Olivia Newton-John Cancer Research Institute, Heidelberg, Victoria 3084, Australia; 3 School of Cancer Medicine, La Trobe University, Bundoora, Victoria 3086, Australia

**Keywords:** apoptosis, BCL-2, BCL-XL, BH3-mimetics, MCL-1

## Abstract

The discovery of a new class of small molecule compounds that target the BCL-2 family of anti-apoptotic proteins is one of the great success stories of basic science leading to translational outcomes in the last 30 years. The eponymous BCL-2 protein was identified over 30 years ago due to its association with cancer. However, it was the unveiling of the biochemistry and structural biology behind it and its close relatives’ mechanism(s)-of-action that provided the inspiration for what are now known as ‘BH3-mimetics’, the first clinically approved drugs designed to specifically inhibit protein–protein interactions. Herein, we chart the history of how these drugs were discovered, their evolution and application in cancer treatment.

## Introduction

The BCL-2 family of proteins consists of functionally opposing, though structurally related proteins [1]. The founding member, BCL-2 was discovered in the mid-1980s due to its association with a chromosomal translocation (t(14;18)) characteristic of blood cancers such as follicular lymphoma [2–5]. However, it was not until 1988 that its true function was discovered as an oncogene that promoted cell survival, rather than cell proliferation, like other known oncogenes at that time [6]. Several other pro-survival proteins (BCL-XL, MCL-1, BCL-W and BFL-1) were later discovered, all related by four regions of sequence homology termed BCL-2 homology (BH1–4) domains [7–10]. These were also found in a subset of proteins with death-promoting functions, namely BAX, BAK and BOK (hereafter, the BAX/BAK proteins) [11–13]. In parallel, a second group of pro-apoptotic proteins (i.e. BAD, BIM, BID, BIK, BMF, NOXA, PUMA, HRK), were also discovered that only possessed the BH3 domain, hence, were termed the BH3-only proteins [14–21].

Biochemical and genetic studies soon revealed a general pathway, now known as the intrinsic apoptotic pathway, by which cells commit suicide in response to diverse stresses (e.g. growth factor depletion, reactive oxygen species, endoplasmic reticulum stress, DNA-damaging chemotherapy). In a healthy cell, BAX/BAK proteins reside in an ‘inactivated’ state, either in the cytosol or bound to pro-survival proteins on the mitochondria [12,22–26]. Following a death stimulus, apoptosis is initiated by transcriptional or post-translational up-regulation of the BH3-only proteins. These bind to the pro-survival proteins and release any bound ‘activated’ BAX/BAK-like proteins, or alternatively, they can bind BAX/BAK directly to induce conformational changes that enable them to oligomerise and subsequently form pores in the mitochondrial outer membrane [27–31], resulting in the release of cytochrome *c* into the cytosol [32]. Cytochrome *c* facilitates oligomerisation of APAF-1 and assembly of the apoptosome, a molecular platform which enables sequential activation of proteolytic caspase enzymes (caspase 9, then caspase 3/7) [33] that cleave important intracellular substrates, leading to the demise of the cell.

In general, apoptosis is restrained by pro-survival proteins sequestering their pro-apoptotic counterparts. When the levels of pro-apoptotic proteins overwhelm the pro-survival molecules, apoptosis ensues. Deregulated apoptosis due to various cellular defects including abnormal expression of pro-survival (and some pro-apoptotic) proteins, is a recognised ‘hallmark’ of most, if not all cancers, as it enables abnormal or damaged cells to survive when they should otherwise be eliminated [34,35]. It also leads to resistance to anti-cancer treatments such as chemotherapy and radiotherapy that predominantly act by activating apoptosis through up-regulation of BH3-only proteins, including via the transcription factor TP53. Inactivating mutations in TP53 are highly prevalent in cancer, leading to drug resistance. Combined, these findings led to the premise that drugs that could mimic the BH3-only proteins, and by-pass the requirement for activation of upstream apoptotic regulators such as TP53, could be highly effective anti-cancer agents. There are now numerous small molecule compounds targeting all BCL-2 pro-survival proteins, either specifically or in combination, except BFL-1 (Table 1). Below, we review how these drugs were discovered and how they have been applied in cancer.

**Table 1.** Key BH3-mimetics leading to the clinical applications or as tool compounds

Compound	Discovery	Notes	Clinical application	References
Pan-specific BCL-2/BCL-XL				
ABT-737	Fragment library screen using SAR-by-NMR	First bona fide BH3-mimetic	No — tool compound	[54]
ABT-263 (Navitoclax)	Modification of three sites on ABT-737 to improve PK properties	First orally bioavailable BH3-mimetic. First BH3-mimetic to enter clinical trials	Yes — clinical trials initially halted due to dose-limiting thrombocytopaenia though now being explored in other cancer types	[55]
AZD4320/AZD0466	Structure-guided modification of Navitoclax to add solubilising moieties	AZD4320 is now formulated as a novel nanoparticle, AZD0466 for clinical application	Yes — trials (solid and haematological). AZD0466 administered intravenously and only induces transient thrombocytopaenia	[71,72]
BCL-XL-specific				
WEHI-539	High thoughput small molecule screen against BCL-W	First potent BCL-XL-specific compound	No — tool compound	[74]
A-115463, A-1331852, A-1293102	Iterative SAR-by-NMR coupled with structure-guided design using WEHI-539 as starting point	A-1331852 was the first orally-available BCL-XL-specific BH3-mimetic with potent *in vivo* activity	No — useful tool compounds for determining tumour BCL-XL dependence	[75,76,77,78]
PZ15227, DT2216	Modification of ABT-263 linking it to E3 ligase ligands for cereblon (PZ15227) and Von Hippel Landau protein (DT2216).	PROTAC forms of ABT-263 that degrade BCL-XL and can overcome thrombocytopaenia as E3 ligases are poorly expressed in platelets. Conversion of ABT-263 to PROTACs makes it more specific for BCL-XL.	No — tool compounds	[132–135]
BCL-2-specific				
ABT-199 (Venetoclax), ABBV-167	Structure-guided design based on Navitoclax	First BCL-2-specific compound. ABBV-167 is a pro-drug form that increases solubility and oral exposure.	Yes — first approved BH3-mimetic. Currently being used for haematological cancers but being trial in various others and solid cancers	[79,83]
S55746/BCL201	Structure-guided design based on literature compound	Potent BCL-2 specific inhibitor	Yes — trials in haematological cancers	[84]
MCL-1-specific				
A-1210477	SAR-by-NMR fragment screen	First MCL-1-specific compound reported with (modest) biological activity	No — tool compound	[102,103]
S63845/MIK665	Fragment-based screen	First MCL-1-specific compound reported with potent *in vivo* activity	Yes — trials of MIK665 in haematological malignancies	[106–108]
AMG-176/AMG-397	High throughput screen plus structure-guided design (AMG-176). Details not reported for AMG-397.	Potent MCL-1 inhibitor with *in vivo* activity. AMG-397 first orally dosed MCL-1 inhibitor to enter trials.	Yes — trials halted for both due to reported cardiotoxicity observed with AMG-397.	[111]
AZD5991	Structure-guided design	Potent MCL-1 inhibitor with *in vivo* activity	Yes trials in haematological malignancies	[110]
sMCL1–2, C3, C5	Modification of MCl-1 inhibitor A-1210477 (sMCL-1–2), or MCL-1/BCL-2 dual inhibitors S1–6 or Nap-1 (C3, C5), linking them to E3 ligase ligand for cereblon	PROTAC forms that induce MCL-1 degradation.	No — tool compounds	[136,137]

## Structures of pro-survival proteins and pro-apoptotic ligands informed BH3-mimetic development

**Figure 1.**
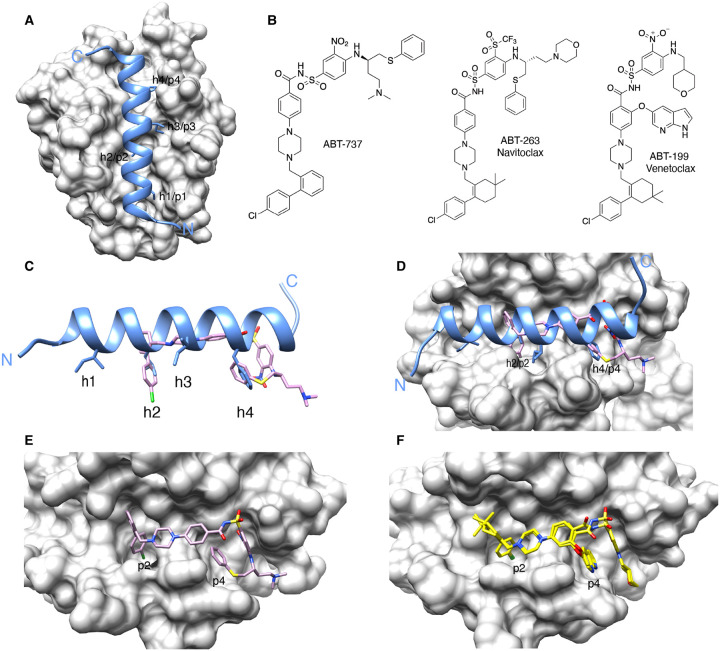
Discovery of the first bona fide BCL-XL/BCL-2 targeting BH3-mimetics. (**A**) Crystal structure of BCL-XL (grey) bound to a peptide corresponding to the BIM BH3 domain (blue; PDB: 3FDL) showing the conserved hydrophobic residues (h1–h4) engaging four pockets (p1–p4) along a large surface groove. All BH3 domains bind to pro-survival proteins similarly. (**B**) Chemical structures showing the evolution of ABT-737 to Navitoclax then Venetoclax. (**C**) Overlay of ABT-737 (pink) with BIM BH3 (blue) showing how the compound mainly targets (**D**) the pockets engaged by the h2 and h4 residues of BCL-XL (grey; PDB: 2YXJ). Close-up of (**E**) ABT-737 (pink) bound to BCL-XL (grey) and (**F**) Venetoclax (yellow) bound to BCL-2 (grey; PDB: 6O0K).

Early biochemical studies demonstrated that the BH3 domains of pro-apoptotic proteins act as ligands for binding pro-survival proteins, though these interactions can be highly selective [24,36,37]. A breakthrough in the development of drugs targeting BCL-2 proteins came in 1997 with the first structure of a pro-survival protein (BCL-XL) bound to a BH3 domain peptide sequence (from BAK) [38]. Here, the BH3 domain formed an amphipathic helix that engaged a large hydrophobic groove on the surface of BCL-XL. Four conserved hydrophobic residues (‘h1–h4’) on one face of the helix penetrated corresponding pockets lining the groove (‘p1–p4’). A conserved aspartate also formed a characteristic salt bridge with an arginine residue, conserved in all pro-survival proteins. Subsequent structures of other pro-survival protein:BH3 domain complexes all possessed the same overall characteristics [39,40] (Figure 1A), providing a prototype for small molecule BCL-2 antagonists that could mimic this common interaction mode to trigger apoptosis.

This review discusses the major successes in the evolution of such ‘BH3-mimetic’ drugs. Numerous other compounds have been labelled as BH3-mimetics, including the widely studied obatoclax and gossypol. Whilst these induce apoptosis, they also affect multiple other pathways (e.g. cell cycle, autophagy, DNA damage response) [41–49], thereby distinguishing themselves from bona fide BH3-mimetics that induce apoptosis in a purely BAX/BAK-dependent fashion [50–52]. Accordingly, these putative BH3-mimetics are not discussed further.

## BH3-mimetics against the major cancer-associated BCL-2 pro-survival proteins

### ABT-737 and ABT-263 — the first bona fide BH3-mimetics targeting BCL-2/BCL-XL

Early attempts at generating compounds that mimic BH3 domain binding interactions included oligoamide ‘foldamers’ that projected hydrophobic moieties similarly to the h1–h4 residues [53]. Although these inhibited BAK BH3 binding to BCL-XL, their affinity was relative weak (low micromolar) and there was no evidence for them possessing cell-killing activity.

In 2005, Abbott Laboratories (now AbbVie), reported ABT-737, an acylsulfonamide compound with low nanomolar affinity for BCL-2, BCL-XL and BCL-W but negligible binding to MCL-1 or BFL-1 [54] (Figure 1B). ABT-737 was discovered using a ‘structure–activity relationships (SAR)-by-NMR’ approach that involved screening BCL-XL against small molecule ‘fragment’ libraries where binders were identified by NMR chemical shift perturbations. Initially, two very weak binders (*K*_d_ of 0.3 mM and 4 mM) were identified engaging the p2 and p4 pockets (Figure 1C,D,E). Subsequent structure-guided medicinal chemistry linked these and enhanced their binding site occupancy, resulting in a compound with single-agent activity on multiple cancer cell lines, and in xenograft models of lymphoma and small cell lung cancer.

Due to its low solubility and poor oral bioavailability, an analogue was later developed by targeting three sites on the ABT-737 backbone that affected charge balance, metabolism, and oral absorption. The resulting compound, ABT-263 (tradename Navitoclax) (Figure 1B), maintained the same target binding profile as ABT-737, but improved its oral absorption and pharmacokinetic properties [55]. Due to promising pre-clinical activity [55,56], Navitoclax entered Phase I/II clinical trials where some efficacy was observed [57–62]. However, patients developed dose-limiting thrombocytopaenia, which was attributed to its potent targeting of BCL-XL, the critical pro-survival protein for platelet survival [55,63]. Nevertheless, Navitoclax is still undergoing Phase I/II clinical trials (particularly in combination with other anti-cancer agents) for various indications including myelofibrosis, melanoma and other advanced solid cancers. It is also the basis for several ‘next-generation’ BH3-mimetics (see below).

### Other BCL-2/BCL-XL dual-specific inhibitors

Since the disclosure of ABT-737/Navitoclax, additional BCL-2/BCL-XL dual inhibitors have been reported, and some are now undergoing clinical evaluation. For example, structure-guided *de novo* design on a benzoylurea scaffold led to nanomolar binders (IC_50_ 20–40 nM) of both BCL-XL and BCL-2 [64,65]. Similarly, a sub-nanomolar inhibitor of BCL-XL/BCL-2 (BM-1197) with *in vivo* activity was developed by computer-aided structure-based design [66–68]. An apparently related compound, APG-1252-M1, is now undergoing clinical trials in solid cancers [69]. Perhaps most promising is AZD4320 based on the ABT-737/Navitoclax scaffold [70]. Through the addition of solubilising moieties guided by structural data, AZD4320 evolved to have similar affinity for BCL-XL/BCL-2 as Navitoclax and equivalent or more potent biological activity [71]. A major advantage of AZD4320 over Navitoclax is it requires just once weekly dosing to be effective which is given by intravenous delivery, rather than daily oral dosing. This reduced exposure only causes transient thrombocytopaenia with platelet levels recovering within 72 h [71], unlike with Navitoclax where platelets are persistently supressed. A novel nanoparticle formulation of AZD4320, AZD0466 [72], is currently undergoing clinical trials (see below).

### BCL-XL-specific BH3-mimetics

Due to the dependence of many cancers, especially solid tumours, on BCL-XL (see accompanying article), there is still considerable interest in developing BCL-XL-targeting agents, despite the potential for thrombocytopaenia. Discriminating BCL-XL from BCL-2, however, is challenging due to their ligand binding grooves being highly conserved between the proteins.

An early attempt to use structural and biochemical data as a basis for increasing the affinity of ABT-737 for MCL-1 inadvertently resulted in a compound (W1191542) with 10-fold selectivity for BCL-XL over BCL-2 [73]. Associated studies showed structural changes upon W1191542 binding to BCL-XL consistent with the binding groove progressively opening along its length. Notably, W1191542 was significantly less active than ABT-737 despite similar affinities for BCL-XL, which was attributed to its faster off-rate.

A more potent BCL-XL-selective compound WEHI-539 emerged from a high-throughput screen originally performed against BCL-W [74]. Hits that engaged the p2 pocket were extended into the p4 pocket guided by extensive structural studies, increasing their affinity and selectivity for BCL-XL over other pro-survival proteins (IC_50_ 1 nM, 400-fold selectivity over BCL-2). The BCL-XL functional specificity of WEHI-539 was confirmed using engineered cell lines and by its potent killing of platelets. Notably, WEHI-539 is considerably smaller than Navitoclax (584 Da versus 975 Da) being based on a different chemical (benzothiazole hydrazone) scaffold.

SAR-by-NMR and structure-guided approaches to replace the labile and potentially toxic hydrazone moiety of WEHI-539 led to A-1155463 which has increased selectivity for BCL-XL over BCL-2 (>1000-fold), increased cell-based activity and modest *in vivo* activity [75]. Further improvements using structure-based design generated A-1331852 with further increased affinity for BCL-XL [76,77] and importantly oral bioavailability, affording it potent anti-tumour activity in haematological and solid tumour mouse models as a single agent, or in combination with chemotherapy. Very recently, AbbVie reported A-1293102, an even more potent and selective BCL-XL inhibitor that combines elements of A-1155463 and ABT-737/Navitoclax [78].

Although A-1331852 or A-1293102 have yet to undergo clinical assessment, they have significant utility as probes for determining tumour pro-survival protein dependency, and/or providing proof-of-principle for BCL-XL targeting in different cancers [76]. Potential avenues to overcome issues such as thrombocytopaenia associated with such agents are discussed below (see ‘Next-generation BH3-mimetics’ section).

### BCL-2-specific BH3-mimetics

As many haematological cancers are predominantly dependent on just BCL-2, there was a strong rationale to develop BCL-2-selective compounds that should obviate the platelet killing that is exclusively associated with BCL-XL inhibition. Accordingly, AbbVie initially attempted to reverse engineer Navitoclax to reduce some of its affinity for BCL-XL. In the process, a co-crystal structure of one analogue with some selectivity towards BCL-2 fortuitously crystallised as a dimer where a tryptophan residue from one monomer crossed into the p4 pocket of the other [79]. Critically, it was noted that the tryptophan's nitrogen atom interacted with Asp103 on BCL-2, one of the few residues not conserved in BCL-XL (it is a glutamate). This interaction was eventually mimicked, and further interactions gained through additional medicinal chemistry on the central core and the p2 pocket binding portion, leading to a potent and highly selective BCL-2 inhibitor, ABT-199 (tradenames Ventoclax/Venetoclaxa) (*K*_i_ 0.01 nM for BCL-2, 48 nM for BCL-XL, 245 nM for BCL-W and >444 nM for MCL-1) [79] (Figure 1B,F).

Venetoclax has potent, single-agent activity against lymphoma and leukaemia cancer cell lines, particularly those with higher BCL-2 levels due to BCL-2 amplification or t(14;18) translocation. Pre-clinical mouse studies showed efficacy without the thrombocytopaenia associated with Navitoclax. A highly promising small initial trial in human chronic lymphocytic leukaemia (CLL) patients [79] led to further successful large-scale clinical trials, and the fast-tracking of Venetoclax for approval for use in patients with CLL (with 17p deletion) in April 2016. It is now approved for, and showing remarkable outcomes in, several haematological cancers including acute myeloid leukaemia and small lymphocytic lymphoma, alone or in combination with other agents such as rituximab and chemotherapy [80–82]. Further trials across multiple malignancies continue, and although death associated with tumour lysis syndrome was observed in a few patients, response rates have been impressive [81]. Very recently, a pro-drug form of Venetoclax (ABBV-167) was developed to increase its solubility and exposure of the parent drug following oral dosing [83]. On the back of these successes, Servier Laboratories developed another BCL-2-selective inhibitor (S55746/BCL201) using structure-guided approaches on a moderately active ‘literature compound’ [84]. *In vitro* and pre-clinical *in vivo* studies demonstrated S55746 is as active as Venetoclax, and is now being trialled in haematological cancers.

One issue that has become apparent with the clinical use of Venetoclax is the development of drug resistance due to mutations emerging that impact the binding site [85–89]. At a structural level, these have only minor effect on the binding mode of Venetoclax, but reduce its affinity for BCL-2 [87]. Although S55746 targets the same general binding site, its binding mode is apparently different, hence, could provide a means for overcoming resistance if it develops with Venetoclax.

### MCL-1-specific BH3-mimetics

Although MCL-1 was the last of the key cancer-associated BCL-2 pro-survival proteins targeted with BH3-mimetics, several potent MCL-1-specific compounds have now been reported. The importance of MCL-1 in cancer was demonstrated in multiple mouse models where *Mcl1* deletion has a profound effect on the initiation and development of haematological cancers [90]. Gene expression analysis across many cancers also showed *Mcl1* is amplified in ∼10% of cases [91]. With the discovery of ABT-737, MCL-1 expression was also demonstrated to be a significant barrier to realising the full potential of BH3-mimetics targeting BCL-2 or BCL-XL [50,92]. Accordingly, various studies showed that targeting MCL-1, either through genetic- or BH3 peptide-based approaches, makes resistant cells significantly more sensitive to BCL-XL/BCL-2 inhibition, and abrogates growth of MCL-1-dependent cancers (e.g. AML and MYC-driven lymphoma) [74,93–96]. These studies provided a strong rationale for the development of MCL-1-targeting BH3-mimetics.

Biochemical mutagenesis studies on BH3 peptides showed MCL-1 was distinct from other pro-survival proteins as it tolerates substitutions at the h2 and h4 positions critical for BCL-XL/BCL-2 binding [96,97], suggesting its binding groove is quite distinct, and providing a rationale for why BCL-2/BCL-XL inhibitors fail to bind MCL-1. Accordingly, screening campaigns were conducted by different groups to identify novel agents against MCL-1. The first to emerge was from the Fesik group [98] using an NMR-based fragment screen. Low affinity (*K*_i _∼ 50–100 µM) fragments of different classes that bound in an overlapping manner in the p2 region were eventually merged, resulting in a final compound with *K*_i_ <100 nM for MCL-1 and significant selectivity over BCL-2/BCL-XL, though no biological activity was reported. The affinity of these compounds was later improved >100-fold (generating VU661013) using structure-based design, whilst maintaining selectivity over BCL-XL/BCL-2, by extending them into the p4 pocket [99–101]. Around the same time, AbbVie performed a small molecule high-throughput screen to identify a hit that eventually became A-1210477 which possessed a similar core to the Fesik compounds, though with higher (sub-nanomolar) affinity [102,103]. Although compromised by high serum protein binding, A-1210477 has modest (micromolar) killing activity in MCL-1-dependent cell lines and potentiates the effects of BCL-XL/BCL-2 inhibitors and other targeted therapies [103–105].

**Figure 2.**
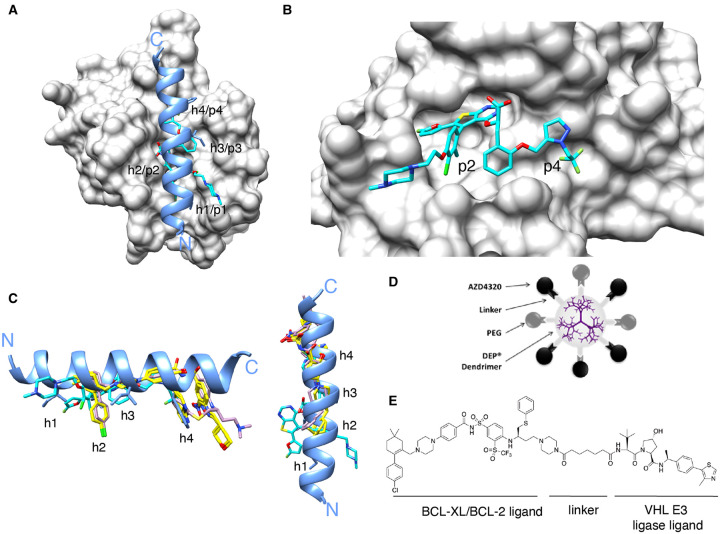
MCL-1 targeting compounds and next-generation BH3-mimetics. (**A**) Structure of a peptide corresponding to the BIM BH3 domain (blue) bound to MCL-1 (grey; PDB: 2NL9) overlayed with S63845 (cyan) from a structure of it in complex with MCL-1 (PDB: 5LOF). (**B**) Close-up of how S63845 (cyan) engages the p2 and p4 pockets of MCL-1 (grey; PDB: 5LOF). Note the interaction at p2 is somewhat different to that of ABT-737 and Venetoclax (see Figure 1E,F). This is further illustrated in (**C**) showing an overlay of the BIM BH3 helix (blue) with S63845 (cyan), ABT-737 (pink) and Venetoclax (yellow). (**D**) Schematic illustrating AZD0466 consisting of Starpharma's polylysine dendrimer and AstraZeneca's BCL-XL/BCL-2 dual inhibitor, AZD4320. (**E**) Chemical structure of the DT2116 PROTAC targeting BCL-XL.

A major breakthrough came with the development of S63845 by Servier [106,107] (Figure 2A,B). This potent and selective MCL-1 inhibitor (*K*_d_ 0.19 nM, 20-fold more potent than A-1210477) was discovered using a fragment-based screen and structure-based design and targets p2 and p4, though somewhat differently than the BCL-X/BCL-2 inhibitors (Figure 2B,C). S63845 potently kills MCL-1-dependent haematological (e.g. multiple myeloma, acute myeloid leukaemia (AML)) and some solid cancers, as a single agent or in combination with other anti-cancer agents. More importantly, S63845 also has robust *in vivo* activity. A related compound S64315/MIK665 is now undergoing clinical trials [108].

Although there are reports of multiple other MCL-1 inhibitors (see [109] for a recent review), the most advanced are AZD5991 (AstraZeneca), a sub-nanomolar MCL-1 macrocyclic inhibitor developed using structure-guided design [110], and AMG-176 (Amgen) [111], an orally bioavailable inhibitor derived from a high-throughput screen coupled with structure-based design. Both compounds have also entered clinical trials in haematological malignancies.

Notably, most MCL-1 inhibitors were well-tolerated in pre-clinical studies. This was not necessarily expected as genetically modified mice with *Mcl1* deletion have defects in several tissues including heart and haematopoietic stem cells [112,113]. One explanation for this is that drug treatment has only a transient effect on MCL-1 *versus* the permanent absence following *Mcl1* deletion. MCL-1-targeting compounds (as seen with some BH3 peptides [114]) also bind mouse MCL-1 weaker than human MCL-1 which perhaps influences outcomes. As such, mice ‘humanised’ for MCL-1 were generated, and although their tumours were more sensitive to S63845, and significant reductions were observed in some blood cell populations following AMG-176 treatment, no major organ toxicities were reported [111,115]. It was, therefore, somewhat surprising that clinical trials on AMG-397 (the first orally dosed MCL-1 inhibitor, also from Amgen; details not yet published) were recently halted due to a ‘cardio signal’. Hence, trial outcomes with other compounds currently under investigation are eagerly awaited as they could significantly influence the future of MCL-1-targeting agents progressing into the clinic.

## Peptides as BCL-2-targeting agents in cancer

Although the major focus around targeting BCL-2 proteins in cancer has been on the development of small molecule BH3-mimetics, there have also been some efforts towards the generation of BH3 peptide-based molecules. This approach is hampered by peptides generally being readily degraded by proteases and unable to cross cell membranes. However, BH3 peptides have proven to be a useful model system for the development of generic strategies to overcome these issues as they are readily assayed, have high affinity for their targets, and have obvious therapeutic potential.

Two main approaches have been applied to BH3 peptides in this context. The first is through the incorporation of hydrocarbon ‘staples’ linking residues several turns apart on the same face of the BH3 helix. Staples are advantageous as they lock BH3 peptides into an α-helical conformation, making them less prone to proteolysis, and they also possess cell-penetrating properties. Early efforts focussed on direct targeting of BAX via pan-specific BID and BIM BH3 domain stapled sequences which notably showed evidence of *in vivo* activity [116,117]. More recently, stapling has been applied to BH3 peptides specific for particular pro-survival proteins including MCL-1 and BFL-1 (currently not targeted by any small molecules) [118–121].

The second approach is to incorporate β-amino acids into BH3 domain sequences as these are not readily recognised by proteases and, therefore, confer protection from proteolysis [122–128]. As β-amino acid incorporation alone does not enable cell entry, these peptides have also been prepared with hydrocarbon staples which has afforded them activity on cancer cells [124]. Despite these successes, peptide-based BCL-2-targeting agents have yet to advance beyond the laboratory.

## Next-generation BH3-mimetics and overcoming toxicities

Probably the biggest limitation associated with current BH3-mimetics is the dose-limiting thrombocytopaenia induced by BCL-XL inhibition. This is a particularly significant issue for the treatment of many solid tumours that are largely BCL-XL-dependent [129]. However, several different tumour-targeting approaches are showing promise for overcoming this problem.

Although intermittent (intravenous) dosing of the dual BCL-XL/BCL-2 inhibitor AZD4320 causes only transient thrombocytopaenia, pre-clinical testing also revealed unexpected cardiotoxicity at sub-efficacious doses [72]. To overcome this issue, AstraZeneca developed a nanomedicine formulation based on Starpharma's DEP® dendrimer PEGylated poly-l-lysine platform. Dendrimers are branched polymers that are smaller than most nanoparticles but with many surface groups for drug conjugation via hydrolysable linkers. Following modelling and testing of different dendrimer formulations, AZD0466 was developed (Figure 2D) [72]. AZD0466 provides prolonged responses in haematological and solid tumour xenografts with only transient thrombocytopaenia and no cardiotoxicity. This improved tolerability is probably due to multiple factors related to the optimised release rate that minimises AZD4320 plasma *C*_max_, and its accumulation at tumour sites [72,130,131]. AZD0466 is now undergoing clinical trials in haematological and solid cancers.

A second approach that has gained recent attention is to use proteolysis-targeting chimera (PROTAC) technology. PROTACs couple a ligand targeting the protein of interest to a second ligand for recruitment of E3 ubiquitin ligases. Hence, PROTAC treatment results in proximity-induced ubiquitination of the target and its subsequent degradation. As this process is catalytic, sub-stoichiometric levels can be highly efficacious, therefore, potentially lower doses can be administered. As different E3 ligases are differentially expressed in different cell types, PROTAC tissue specificity can potentially be ‘tuned’. For example, PROTACs PZ15227 and DT2216 (Figure 2E) linking ABT-263 to E3 ligase ligands for cereblon or Von Hippel Landau protein, respectively, effectively degrade BCL-XL in a range of cell types, but not platelets where both E3 ligases are poorly expressed [132–135]. Moreover, they demonstrate more potent tumour cell-killing activity than ABT-263 *in vitro* and *in vivo*. Interestingly, linkage of ABT-263 within the PROTAC lowered its affinity for BCL-2, hence, it does not induce BCL-2 degradation. Several MCL-1 PROTACs (based on A-1210477, S1–6 and Nap-1) have also been reported [136,137] which potentially could prove useful for overcoming the cardiotoxicity noted above.

One final approach to making BH3-mimtics more specific for tumour cells is by linking them to antibodies directed against antigens preferentially expressed on tumours (e.g. mutant EGF receptor). Reports of antibody-drug conjugates (ADCs), such as with A-1331852, have only appeared in the patent literature, however, the supporting data presented suggests they maintain anti-tumour activity whilst sparing platelets.

One further issue with targeting BCL-XL in some cancers, especially solid tumours, is that co-targeting of MCL-1 is required for maximal killing activity [129]. However, as some normal tissues (such as liver) are highly dependent on BCL-XL *plus* MCL-1 for their survival, fatal toxicity has been observed in mice upon co-administration of MCL-1 and BCL-XL inhibitors [129]. Whilst there is evidence this can be prevented by careful dose scheduling [138], the inherent dangers this presents for clinical application means that more tissue-specific approaches (e.g. ADCs or even PROTACs) might also have greater utility for extending the range of cancers that can be treatable with BH3-mimetics.

## Concluding remarks

The success of Venetoclax in the clinic has undeniably validated BH3-mimetics for cancer treatment. However, the fact that it is currently the only clinically approved BH3-mimetic, despite multiple other compounds showing promising pre-clinical activity, emphasises how the full potential of BH3-mimetics has yet to be achieved. Additionally, the emergence of resistance mechanisms as well as unknown toxicities are significant challenges that must be addressed in the next phase of progressing any new BH3-mimetic compounds towards the clinic. Nevertheless, exciting new approaches such as PROTACs, ADCs and novel formulations show enormous potential for overcoming many of these issues, making it likely that BH3-mimetics will soon have significant impact for the treatment of a wider range of cancers in the future.

**Perspective**

- BH3-mimetic drugs represent the first clinically approved compounds developed to target protein:protein interactions. They are now having a significant impact in patients with haematological malignancies.
- Currently, there are multiple BH3-mimetics against different BCL-2 family targets being developed and trialled for a range of cancers.
- In the future, new approaches, including novel formulations and targeting strategies, will be needed to overcome any on-target toxicities associated with BH3-mimetics, and resistance mechanisms that are emerging.

## Open Access

Open access for this article was enabled by the participation of La Trobe University in an all-inclusive *Read & Publish* pilot with Portland Press and the Biochemical Society under a transformative agreement with CAUL.

## Abbreviations

BAK: BCL-2 antagonist/killer
BAX: BCL-2-associated X protein
BCL-2: B-cell lymphoma 2
BCL-XL: B-cell lymphoma-extra large
BFL-1: Bcl-2-related gene expressed in foetal liver
BH: BCL-2 homology domain
CLL: chronic lymphocytic leukaemia
MCL-1: myeloid cell leukaemia-1
SAR-by-NMR: structure activity relationships by nuclear magnetic resonance
